# Supplementary material for: HIVprotI: an integrated web based platform for prediction and design of HIV proteins inhibitors
Source: J Cheminform. 2018 Mar 9;10:12. doi: 10.1186/s13321-018-0266-y (PMC5845081; doi:10.1186/s13321-018-0266-y)
Supplement: Supplementary file 2 — Additional file 2. Source code of HIVProtI web server. [file 13321_2018_266_MOESM2_ESM.zip › HIVprotI_Source-code/hivprotI/weka-3-6-6/doc/weka/classifiers/trees/j48/GraftSplit.html]

GraftSplit


---


|  |  |  |
| --- | --- | --- |
| **Overview** \| **Package** \| **Class** \| **Tree** \| **Deprecated** \| **Index** \| **Help** \| **Weka's home** | |  |
| **PREV CLASS**   **NEXT CLASS** | **FRAMES**    **NO FRAMES**     **All Classes** |  |
| SUMMARY: NESTED \| FIELD \| CONSTR \| METHOD | DETAIL: FIELD \| CONSTR \| METHOD |  |


---


## weka.classifiers.trees.j48 Class GraftSplit

```
java.lang.Object
  weka.classifiers.trees.j48.ClassifierSplitModel
      weka.classifiers.trees.j48.GraftSplit
```

**All Implemented Interfaces:**: java.io.Serializable, java.lang.Cloneable, java.lang.Comparable, RevisionHandler

---

``` public class GraftSplit extends ClassifierSplitModel implements java.lang.Comparable ```

Class implementing a split for nodes added to a tree during grafting.

**Version:**
:   $Revision 1.0 $

**Author:**
:   Janice Boughton (jrbought@infotech.monash.edu.au)

**See Also:**: Serialized Form

---

**Constructor Summary**

`GraftSplit(int a,
double v,
int t,
double c,
double l)`
  
          constructor

`GraftSplit(int a,
double v,
int t,
double oC,
double[][] counts)`
  
          constructor


| **Method Summary** | |
| --- | --- |
| `int` | `attribute()` |
| `void` | `buildClassifier(Instances data)`             builds m\_graftdistro using the passed data |
| `double` | `classProb(int classIndex, Instance instance, int theSubset)`             returns the probability for instance for the specified class |
| `int` | `compareTo(java.lang.Object g)`             method needed for sorting a collection of GraftSplits by laplace value |
| `void` | `deleteGraftedCases(Instances data)`             deletes the cases in data that belong to leaf pointed to by the test (i.e. |
| `java.lang.String` | `dumpLabelG(int index, Instances data)`             Prints label for subset index of instances (eg class). |
| `NoSplit` | `getLeaf()` |
| `NoSplit` | `getOtherLeaf()` |
| `java.lang.String` | `getRevision()`             Returns the revision string. |
| `double` | `laplaceForSubsetOfInterest()` |
| `java.lang.String` | `leftSide(Instances data)`             Prints left side of condition satisfied by instances. |
| `int` | `maxClassForSubsetOfInterest()` |
| `double` | `positives(int subset)` |
| `double` | `positivesForSubsetOfInterest()` |
| `java.lang.String` | `rightSide(int index, Instances data)`             Prints condition satisfied by instances in subset index. |
| `java.lang.String` | `sourceExpression(int index, Instances data)`             Returns a string containing java source code equivalent to the test made at this node. |
| `double` | `splitPoint()` |
| `int` | `subsetOfInterest()` |
| `int` | `testType()`             returns the test type |
| `java.lang.String` | `toString(Instances data)`             method for returning information about this GraftSplit |
| `double` | `totalForSubset(int subset)` |
| `double` | `totalForSubsetOfInterest()` |
| `double[]` | `weights(Instance instance)`             Returns weights if instance is assigned to more than one subset. |
| `int` | `whichSubset(Instance instance)`             Returns index of subset instance is assigned to. |

**Methods inherited from class weka.classifiers.trees.j48.ClassifierSplitModel**

`checkModel, classifyInstance, classProbLaplace, clone, codingCost, distribution, dumpLabel, dumpModel, numSubsets, resetDistribution, sourceClass, split`

**Methods inherited from class java.lang.Object**

`equals, getClass, hashCode, notify, notifyAll, toString, wait, wait, wait`

**Constructor Detail**

### GraftSplit

```
public GraftSplit(int a,
                  double v,
                  int t,
                  double c,
                  double l)
```

:   constructor

    **Parameters:**: `a` - the attribute to split on: `v` - the value of a where split occurs: `t` - the test type (0 is <=, 1 is >, 2 is =, 3 is !): `c` - the class to label the leaf node pointed to by test as.: `l` - the laplace value (needed when sorting GraftSplits)

---


### GraftSplit

```
public GraftSplit(int a,
                  double v,
                  int t,
                  double oC,
                  double[][] counts)
           throws java.lang.Exception
```

:   constructor

    **Parameters:**: `a` - the attribute to split on: `v` - the value of a where split occurs: `t` - the test type (0 is <=, 1 is >, 2 is =, 3 is !=): `oC` - the class to label the leaf node not pointed to by test as.: `counts` - the distribution for this split **Throws:**: `java.lang.Exception`


**Method Detail**

### deleteGraftedCases

```
public void deleteGraftedCases(Instances data)
```

:   deletes the cases in data that belong to leaf pointed to by
    the test (i.e. the subset of interest). this is useful so
    the instances belonging to that leaf aren't passed down the
    other branch.

    :   **Parameters:**: `data` - the instances to delete from

---


### buildClassifier

```
public void buildClassifier(Instances data)
                     throws java.lang.Exception
```

:   builds m\_graftdistro using the passed data

    :   **Specified by:**: `buildClassifier` in class `ClassifierSplitModel`
    :   **Parameters:**: `data` - the instances to use when creating the distribution **Throws:**: `java.lang.Exception` - if something goes wrong

---


### getLeaf

```
public NoSplit getLeaf()
```

:   **Returns:**: the NoSplit object for the leaf pointed to by m\_testType branch

---


### getOtherLeaf

```
public NoSplit getOtherLeaf()
```

:   **Returns:**: the NoSplit object for the leaf not pointed to by m\_testType branch

---


### dumpLabelG

```
public final java.lang.String dumpLabelG(int index,
                                         Instances data)
                                  throws java.lang.Exception
```

:   Prints label for subset index of instances (eg class).

    :   **Parameters:**: `index` - the bag to dump label for: `data` - to get attribute names and such **Returns:**: the label as a string **Throws:**: `java.lang.Exception` - if something goes wrong

---


### subsetOfInterest

```
public int subsetOfInterest()
```

:   **Returns:**: the subset that is specified by the test type

---


### positivesForSubsetOfInterest

```
public double positivesForSubsetOfInterest()
```

:   **Returns:**: the number of positive cases in the subset of interest

---


### positives

```
public double positives(int subset)
```

:   **Parameters:**: `subset` - the subset to get the positives for **Returns:**: the number of positive cases in the specified subset

---


### totalForSubsetOfInterest

```
public double totalForSubsetOfInterest()
```

:   **Returns:**: the number of instances in the subset of interest

---


### totalForSubset

```
public double totalForSubset(int subset)
```

:   **Parameters:**: `subset` - the index of the bag to get the total for **Returns:**: the number of instances in the subset

---


### leftSide

```
public java.lang.String leftSide(Instances data)
```

:   Prints left side of condition satisfied by instances.

    :   **Specified by:**: `leftSide` in class `ClassifierSplitModel`
    :   **Parameters:**: `data` - the data.

---


### attribute

```
public int attribute()
```

:   **Returns:**: the index of the attribute to split on

---


### rightSide

```
public final java.lang.String rightSide(int index,
                                        Instances data)
```

:   Prints condition satisfied by instances in subset index.

    :   **Specified by:**: `rightSide` in class `ClassifierSplitModel`

---


### sourceExpression

```
public final java.lang.String sourceExpression(int index,
                                               Instances data)
```

:   Returns a string containing java source code equivalent to the test
    made at this node. The instance being tested is called "i".

    :   **Specified by:**: `sourceExpression` in class `ClassifierSplitModel`
    :   **Parameters:**: `index` - index of the nominal value tested: `data` - the data containing instance structure info **Returns:**: a value of type 'String'

---


### weights

```
public double[] weights(Instance instance)
```

:   **Description copied from class: `ClassifierSplitModel`**
:   Returns weights if instance is assigned to more than one subset.
    Returns null if instance is only assigned to one subset.

    :   **Specified by:**: `weights` in class `ClassifierSplitModel`
    :   **Parameters:**: `instance` - the instance to produce the weights for **Returns:**: a double array of weights, null if only belongs to one subset

---


### whichSubset

```
public int whichSubset(Instance instance)
```

:   **Description copied from class: `ClassifierSplitModel`**
:   Returns index of subset instance is assigned to.
    Returns -1 if instance is assigned to more than one subset.

    :   **Specified by:**: `whichSubset` in class `ClassifierSplitModel`
    :   **Parameters:**: `instance` - the instance for which to determine the subset **Returns:**: an int indicating the subset this instance belongs to

---


### splitPoint

```
public double splitPoint()
```

:   **Returns:**: the value of the split point

---


### maxClassForSubsetOfInterest

```
public int maxClassForSubsetOfInterest()
```

:   **Returns:**: the dominate class for the subset of interest

---


### laplaceForSubsetOfInterest

```
public double laplaceForSubsetOfInterest()
```

:   **Returns:**: the laplace value for maxClass of subset of interest

---


### testType

```
public int testType()
```

:   returns the test type

    :   **Returns:**: value of testtype

---


### compareTo

```
public int compareTo(java.lang.Object g)
```

:   method needed for sorting a collection of GraftSplits by laplace value

    :   **Specified by:**: `compareTo` in interface `java.lang.Comparable`
    :   **Parameters:**: `g` - the graft split to compare to this one **Returns:**: -1, 0, or 1 if this GraftSplit laplace is <, = or > than that of g

---


### classProb

```
public final double classProb(int classIndex,
                              Instance instance,
                              int theSubset)
                       throws java.lang.Exception
```

:   returns the probability for instance for the specified class

    :   **Overrides:**: `classProb` in class `ClassifierSplitModel`
    :   **Parameters:**: `classIndex` - the index of the class: `instance` - the instance to get the probability for: `theSubset` - the subset **Throws:**: `java.lang.Exception` - if something goes wrong

---


### toString

```
public java.lang.String toString(Instances data)
```

:   method for returning information about this GraftSplit

    :   **Parameters:**: `data` - instances for determining names of attributes and values **Returns:**: a string showing this GraftSplit's information

---


### getRevision

```
public java.lang.String getRevision()
```

:   Returns the revision string.

    :   **Specified by:**: `getRevision` in interface `RevisionHandler`
    :   **Returns:**: the revision


---


|  |  |  |
| --- | --- | --- |
| **Overview** \| **Package** \| **Class** \| **Tree** \| **Deprecated** \| **Index** \| **Help** \| **Weka's home** | |  |
| **PREV CLASS**   **NEXT CLASS** | **FRAMES**    **NO FRAMES**     **All Classes** |  |
| SUMMARY: NESTED \| FIELD \| CONSTR \| METHOD | DETAIL: FIELD \| CONSTR \| METHOD |  |


---
